# The Up-regulation of TNF-α Maintains Trigeminal Neuralgia by Modulating MAPKs Phosphorylation and BKCa Channels in Trigeminal Nucleus Caudalis

**DOI:** 10.3389/fncel.2021.764141

**Published:** 2021-11-25

**Authors:** Zhan-ying Lu, Juan Fan, Li-hua Yu, Bei Ma, Li-ming Cheng

**Affiliations:** ^1^Experimental Training Center of Basic Medical Science, Naval Medical University, Shanghai, China; ^2^Key Laboratory of Spine and Spinal Cord Injury Repair and Regeneration of the Ministry of Education, Division of Spine Surgery, Department of Orthopedics, Tongji Hospital Affiliated to Tongji University School of Medicine, Shanghai, China

**Keywords:** trigeminal neuralgia, TNF-α, trigeminal nucleus caudalis, MAPKs phosphorylation, BK_*Ca*_ channel

## Abstract

Trigeminal neuralgia (TN) is a severe chronic neuropathic pain. Despite numerous available medical interventions, the therapeutic effects are not ideal. To control the pain attacks, the need for more contemporary drugs continues to be a real challenge. Our previous study reported that Ca^2+^-activated K^+^ channels (BK_*Ca*_) channels modulated by mitogen-activated protein kinases (MAPKs) in the trigeminal ganglia (TG) neurons play crucial roles in regulating TN, and some research studies demonstrated that inflammatory cytokine tumor necrosis factor alpha (TNF-α) could promote neuropathic pain. Meanwhile, the trigeminal nucleus caudalis (TNC), the first central site of the trigeminal nociceptive pathway, is responsible for processing sensory and pain signals from the peripheral orofacial area. Thus, this study is aimed to further investigate whether TNF-α and MAPKs phosphorylation in the TNC could mediate the pathogenesis of TN by modulating BK_*Ca*_ channels. The results showed that TNF-α of the TNC region is upregulated significantly in the chronic constriction injury of infraorbital nerve (ION-CCI) rats model, which displayed persistent facial mechanical allodynia. The normal rats with target injection of exogenous TNF-α to the fourth brain ventricle behaved just like the ION-CCI model rats, the orofacial mechanical pain threshold decreased clearly. Meanwhile, the exogenous TNF-α increased the action potential frequency and reduced the BK_*Ca*_ currents of TNC neurons significantly, which could be reversed by U0126 and SB203580, the inhibitors of MAPK. In addition, U0126, SB203580, and another MAPK inhibitor SP600125 could relieve the facial mechanical allodynia by being injected into the fourth brain ventricle of ION-CCI model rats, respectively. Taken together, our work suggests that the upregulation of TNF-α in the TNC region would cause the increase of MAPKs phosphorylation and then the negative regulation of BK_*Ca*_ channels, resulting in the TN.

## Introduction

Trigeminal neuralgia (TN) is a severe chronic neuropathic pain, which often results from a primary lesion or dysfunction of the nervous system (Jensen et al., 2011). The common symptoms are allodynia, hyperalgesia, and spontaneous pain (Cao et al., 1996; Arch et al., 1998; Chen et al., 2011). Although there are various therapeutic drugs and measures (Montano et al., 2015), the effects are not ideal and a number of patients could develop drug resistance clinically. To control the pain attacks, the need for more contemporary drugs continues to be a real therapeutic challenge. TN cues are detected by nociceptive trigeminal ganglia (TG) neurons and relayed to the trigeminal nucleus caudalis (TNC), which is the first central site of the trigeminal nociceptive pathway and responsible for processing sensory and pain signals from the peripheral orofacial area (Youn, 2014a; Zhang et al., 2015). However, the mechanism of TNC regulating trigeminal neuropathic pain remains to be unclear.

Microglia and astrocytes, two spinal cord glial cells, are important in the establishment and maintenance of neuropathic pain (DeLeo et al., 2004; Tsuda et al., 2005; Lu et al., 2014; Yamamoto et al., 2015). Cytokines tumor necrosis factor alpha (TNF-α), interleukin-1β (IL-1β), and chemokine C–C motif chemokine ligand 2 (CCL2) are essential for glial cell activation, inflammatory pain, and neuropathic pain after tissue or nerve damage (DeLeo and Yezierski, 2001; Scholz and Woolf, 2007; Gao et al., 2009; Mei et al., 2011; Pu et al., 2013). There are some studies showed that the primary culture of astrocytes with TNF-α induced a marked increase of several chemokines, such as CCL2 and CXCL1, which could contribute to central sensitization and chronic pain (Gao et al., 2009). It has been shown that target inhibiting glia-expressing signaling molecules, such as TNF-α, IL-1β, and CCL2, could alleviate neuropathic pain (Milligan, Twining et al., 2003; Wei et al., 2008; Gao et al., 2009).

Mitogen-activated protein kinases (MAPKs) are important biomolecules, which include extracellular signal-regulated kinase (ERK), p38, and c-Jun N-terminal kinase (JNK). The molecules are often involved in the intracellular transduction cascade (Ji et al., 2002; Zhuang et al., 2006). In addition, accumulating evidence has shown that MAPKs (ERK, p38, and JNK) could be activated in spinal astrocytes or microglia after tissue inflammation, nerve injury, or artificial TNF-α stimulation (Ji et al., 2002; Jin et al., 2003; Zhuang et al., 2006), leading to the generation of neuropathic pain by releasing chemokines CXCL1 (Cao et al., 2014) or CCL2 (Gao et al., 2009; Komatsu et al., 2011). Inhibiting ERK, p38, or JNK could effectively alleviate inflammatory and neuropathic pain (White et al., 2011; Crown, 2012). Combined with the previous elaboration, we wonder whether the proinflammatory cytokines (TNF-α and IL-1β) and chemokines (CCL2 and CXCL1) are involved in the regulation of TN in combination with the MAPKs in the TNC region, and what is the molecular mechanism in the pathway.

Large conductance Ca^2+^-activated K^+^ channels (BK_*Ca*_ channels) are characterized by a large single-channel conductance (>100 pS), which could be activated by the synergistic interaction of elevated intracellular Ca^2+^ and electrical depolarization of the membrane, leading to rapid efflux of K^+^ (Rothberg, 2012). BK_*Ca*_ channels expressed in sensory neurons are important regulators of neuron excitability (Sah and Faber, 2002; Wulf-Johansson et al., 2009; Zhang et al., 2010). For example, IbTX, a BK_*Ca*_ channels inhibitor, could significantly increase the spontaneous excitatory postsynaptic currents (sEPSCs) in the superficial dorsal horn and thus modulate the nociceptive transmission (Furukawa et al., 2008). In the ION-CCI model rats, BK_*Ca*_ channels of peripheral TG neurons were identified to be a new therapeutic target for the treatment of TN (Liu et al., 2015). Thus, the aim of this study is to further investigate whether BK_*Ca*_ channels perform a similar function in the TNC region and the potential mechanism.

## Materials and Methods

### Experimental Animals

Male Sprague-Dawley rats weighing 200–250 g were used in experiments. Animal care was consistent with the guidelines set by the Laboratory Animal Center of Naval Medical University. Rats were randomly divided into two groups: the ION-CCI group receiving a CCI of the infraorbital branch of the trigeminal nerve and the sham group receiving the same surgical procedure without constriction. All experimental procedures were approved by the Naval Medical University Institutional Animal Care and Use Committee.

### Chronic Constriction Injury of Infraorbital Nerve and Sham Operation

The unilateral ligation of the ION was conducted according to the method described previously (Liu et al., 2015). Firstly, the SD rats were anesthetized with sodium pentobarbital (50 mg/kg i.p.), and the incision was conducted at the juncture between the zygomatic arch and nasal bone to expose the infraorbital branch of the trigeminal nerve. Then two chromic catgut (4-0) ligations were loosely tied around the nerve with about 2 mm spacing. To obtain the desirable constriction degree, a formulated criterion was applied: the ligatures would reduce the diameter of the ION significantly with no interruption of the circulation through the superficial vasculature. Finally, the incision was sutured by silk sutures (4-0) carefully. In the corresponding sham group, the operation was performed in the same manner except that the ION was not ligated. All operations were performed aseptically and no antibiotics were administered.

### Behavior Testing

The mechanical pain threshold was tested 1 day before and the 15th day after surgery in the two groups. The rats were weighted and placed individually in a stainless-steel cage (20 × 20 × 20 cm) and allowed to acclimatize for at least 1 hour before the test. The mechanical pain threshold was determined with a graded series of weight von Frey filaments (Institute of Autonomic Neuroscience, London, United Kingdom), which could produce a bending force ranging from 0.51 to 61 g. The mechanical stimuli were focused within the ION territory and vibrissa pad with an ascending order of intensity. The pain response threshold was the lowest force of filaments that produced a brisk head withdrawal, touching or scratching the facial regions upon mechanical stimulation and the threshold was quantified in grams. The mechanical test was measured every 10 min following drug administration. The experimenters operating the behavioral measurements were blind to the treatments.

### Enzyme-Linked Immunosorbent Assay

Tumor necrosis factor α, IL-1β, IL-6, IL-10, IL-18, CCL2, and CXCL1 ELISA kits for rats were purchased from Westang Biotechnology Company (China). TNC tissues of sham and ION-CCI rats were collected on the 15th day after surgery and then homogenized in a lysis buffer containing protease and phosphatase inhibitors (Life technology). Protein concentrations were tested by a BCA Protein Assay (Life technology). For each reaction in a 96-well plate, 100 μg proteins were used, and ELISA was performed according to protocol of the manufacturer. The standard curve was included in each experiment.

### Immunofluorescence Histochemistry

The unilateral ligation of the ION was conducted according to the method described previously (Liu et al., 2015). Firstly, the SD rats were anesthetized with sodium pentobarbital (50 mg/kg i.p.), and the incision was conducted at the juncture between the zygomatic arch and nasal bone to expose the infraorbital branch of the trigeminal nerve. Then two chromic catgut (4-0) ligations were loosely tied around the nerve with about 2 mm spacing. To obtain the desirable constriction degree, a formulated criterion was applied: the ligatures would reduce the diameter of the ION significantly with no interruption of the circulation through the superficial vasculature. Finally, the incision was sutured by silk sutures (4-0) carefully. In the corresponding sham group, the operation was performed in the same manner except that the ION was not ligated. All operations were performed aseptically and no antibiotics were administered.

### Western Blotting Analysis

On the postoperative day 15, the TNC regions were harvested and homogenized in cold lysis buffer (20 mM hydroxyethyl piperazineethanesulfonic acid (HEPES), 10 mM KCl, 1.5 mM MgCl_2_, 1 mM ethylenediamine tetraacetic acid (EDTA), 1 mM ethylene glycol tetraacetic acid (EGTA), 1 mM dithiothreitol (DTT), 0.1 mM phenylmethylsulfonyl fluoride (PMSF), 5 mg/mL pepstatin A, 10 mg/mL leupeptin, and 10 mg/ml aprotinin, pH 7.4) using homogenizer. Protein concentration was determined with a BCA assay kit (Life technology, United States) and then heated to 95°C for 10 min. Proteins were separated using sodium dodecyl sulfate polyacrylamide gel electrophoresis (SDS-PAGE) at 300 mA for 90 min and electrophoretically transferred to nitric acid fiber membrane at 100 V for 100 min in Towbin transfer buffer. The transferred membranes were blocked with 5% (mass/vol) non-fat dried milk in Tri-buffered saline containing 0.05% Tween 20 (TBST) for 1 h and then incubated over night at 4°C with the primary antibodies: BK_*Ca*_ (rabbit, 1:1000, Alomone, Israel), ERK1/2 (rabbit, 1:1,000, Cell Signaling Technology, San Antonio, TX, United States), p-ERK1/2 (rabbit, 1:1,000, Cell Signaling Technology, San Antonio, TX, United States), p38 (rabbit, 1:1,000, Cell Signaling Technology, San Antonio, TX, United States), p-p38 (rabbit, 1:1,000, Cell Signaling Technology, San Antonio, TX, United States), JNK (rabbit, 1:1,000, Cell Signaling Technology, San Antonio, TX, United States), p-JNK (mouse, 1:1,000, Cell Signaling Technology, San Antonio, TX, United States), or β-actin (mouse, 1:8,000, Sigma, Burlington, MA, United States). After washing with TBST for 3 times, the membranes were incubated with the secondary antibody respectively for BK_*Ca*_, ERK1/2, p-ERK1/2, p-p38, p38, JNK (HRP-goat anti-rabbit polyclonal, 1:1,000, Proteintech, Chicago, IL, United States), p-JNK, and β-actin (HRP-goat anti-mouse polyclonal, 1:1,000, Proteintech, Chicago, IL, United States) at room temperature for 1 h. The membranes were rinsed in TBST for 5 times at room temperature and then visualized and analyzed using an Amersham Imager 600 Chemiluminescence Imaging System (GE, Boston, MA, United States).

### The Fourth Brain Ventricle Injections

We carried out the fourth brain ventricle injection via foramen magnum as described previously on postoperative day 15, and the drug could follow the cerebrospinal fluid circulation to the TNC region. Briefly, we controlled the conscious rats to shave their hair on the head, and then the rats were anesthetized with gas anesthetic isoflurane. The sterile stainless-steel needle was inserted into the foramen magnum through the cerebral dura mater to the brain ventricle. Then the corresponding drugs were injected slowly (10 μl/min). The mechanical pain threshold was tested every 10 min after drug administration.

### Preparation of Horizontal Brain Stem Slices

Horizontal brain-stem slices (400 μm) were prepared from male Sprague-Dawley rats weighing 100–120 g as described previously (Youn, 2014b). The rats were anesthetized deeply with isoflurane and then decollated. The head was immediately transferred to ice-cold artificial cerebral spinal fluid (ACSF) solution (in mM: NaCl 117, KCl 3.6, CaCl_2_⋅2H_2_O 2.5, MgCl_2_⋅6H_2_O 1.2, D-glucose 11, NaHCO_3_ 25, NaH_2_PO_4_ 1.2; pre-oxygenated with 95% O_2_ and 5% CO_2_; pH 7.4) to remove the cervical spinal cord and vessel fascia, and then the brain stem was taken out carefully. The slices were cut at brain stem −14.08 to −14.6 mm from bregma using a Vibratome 1000 plus (Vibratome, St. Louis, MO, United States) and then incubated at 37°C for at least 1 h. All the processes were oxygenated with 95% O_2_ and 5% CO_2_.

### Whole-Cell Patch-Clamp Recording

All the whole-cell patch-clamp recordings were performed at room temperature with an Axon patch 700B amplifier (Axon Instruments, Burlingame, CA, United States). Patch pipettes had a resistance of 8–10 MΩ, which were made of borosilicate glass capillaries, and the series resistance was compensated at 60% during recording. The signals were digitized at 10–50 kHz and filtered at 2 kHz with holding potential of −60 mV. The pipette solution was as follows (in mM) 120 K gluconate, 10 KCl, 5 NaCl, 2 MgCl_2_⋅6H_2_O, 1 CaCl_2_⋅2H_2_O, 10 HEPES, 11 EGTA, 2 Mg-ATP, 1 Li-GTP, with pH adjusted to 7.4 by KOH. The external solution ACSF contained (in mM) 117 NaCl, 3.6 KCl, 2.5 CaCl_2_⋅2H_2_O, 1.2 MgCl_2_⋅6H_2_O, 11 D-glucose, 25 NaHCO_3_, and 1.2 NaH_2_PO_4_ with pH adjusted to 7.4 by NaOH. The action potentials were recorded with a 700 ms positive current injection ranging from −40 to + 450 pA. The threshold current was identified with a single action potential began to be evoked, and the final action potential frequencies were recorded at the double threshold currents.

The BK_*Ca*_ currents were recorded with the protocol containing a pre-pulse (0 mV and 100 ms) and a followed 400 ms test pulses from − 80 to + 80 mV with a 10 mV increment. The BK_*Ca*_ currents solution contained (in mM) 145 NMDG, 3 KCl, 0.6 MgCl_2_⋅6H_2_O, 2.5 CaCl_2_⋅2H_2_O, 10 HEPES, 10 glucose with pH adjusted to 7.4 by tris-base. Apamin (200 nM) and 4-AP (10 mM) were routinely added to extracellular solutions to minimize interference by small conductance calcium-activated K^+^ (SK) currents and voltage-dependent K^+^ (K_*V*_) currents.

### Drugs

4-aminopyridine (4-AP, a selective blocker of members of Kv1 (KCNA) family of voltage-activated K^+^ channels), Apamin (an inhibitor of SK channels), NMDG (a selective blocker of Na^+^ channels), U0126 (a highly selective inhibitor of ERK kinase), SB203580 (an inhibitor of p38), and SP600125 (an inhibitor of JNK) were all purchased from Sigma.

### Statistical Analysis

The data were shown as mean ± SEM. All statistical analyses were performed using Independent-Samples *T* Test or Paired Student’s *t*-test (SPSS 16.0 version). Asterisks (*) indicated statistically significant differences from the control group (*P < 0.05, ^**^P < 0.01).

## Results

### The Expression of TNF-α, CCL2, and CXCL1 in the Trigeminal Nucleus Caudalis of Chronic Constriction Injury of Infraorbital Nerve Rats

**FIGURE 1:**
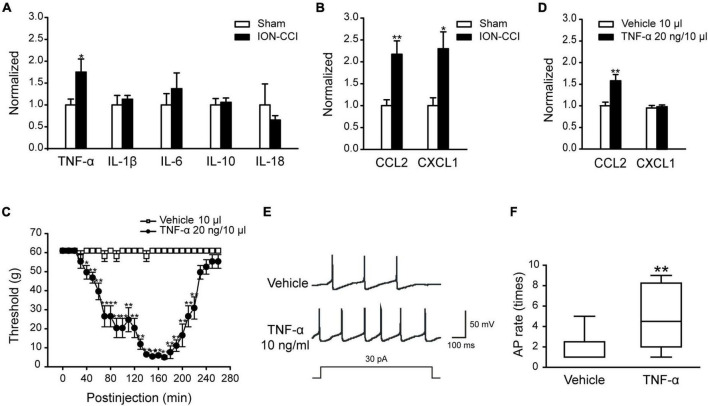
The expressions of some cytokines and chemokines are increased in the TNC region of ION-CCI rats and exogenous TNF-α could induce similar responses like the ION-CCI model and increase the excitability of TNC neurons. **(A)** TNF-α, but not IL-1β, IL-6, IL-10, or IL-18, is increased significantly in the TNC region of ION-CCI rats on the postoperative day 15 compared with the sham group rats, *p < 0.05, *n* = 8. **(B)** Both of CXCL1 and CCL2 are increased significantly in the TNC of ION-CCI rats, *p < 0.05, **p < 0.01, *n* = 8. **(C)** The facial mechanical pain threshold is reduced significantly from 40th to 220th min after injection of exogenous TNF-α (20 ng and 10 μl) to the fourth brain ventricle of normal rats, *p < 0.05, **p < 0.01, *n* = 6. **(D)** At the 150th min after exogenous TNF-α injection, the expression of CCL2 is increased in the TNC, whereas CXCL1 is relatively unchanged, **p < 0.01, *n* = 6. **(E,F)** TNF-α (10 ng/ml) pre-incubated for 1 min could increase the frequency of action potential of TNC neurons significantly with the stimulation of double threshold currents, **p < 0.01, *n* = 10. TNF-α, tumor necrosis factor alpha; TNC, trigeminal nucleus caudalis; ION-CCI, chronic constriction injury of infraorbital nerve; CCL2, C–C motif chemokine ligand 2.

Multiple studies have shown that the inflammatory cytokine TNF-α and chemokine CCL2 are upregulated and involved in the enhancement of neuropathic pain after tissue or nerve damage. In this study, we also found that the expressions of TNF-α, CCL2, and CXCL1 were increased significantly in the TNC of ION-CCI rats on the postoperative day 15 compared with the sham group (Figures 1A,B, *p < 0.05, ^**^p < 0.01, *n* = 8), and TNF-α was largely co-localized with NeuN (marker of neuron) and Iba 1 (marker of microglia), but not GFAP (data not shown). In contrast, the expression of other important cytokines, for example IL-1β, IL-6, IL-10, and IL-18, did not change significantly (Figure 1A).

### The Effects of TNF-α on the Trigeminal Neuralgia

To verify the effect of TNF-α, we directly injected TNF-α into the fourth brain ventricle of normal rats to examine whether the ION-CCI condition could be evoked. The facial mechanical pain threshold was measured every 10 min before and after TNF-α administration. The results showed that TNF-α (20 ng, 10 μl) could significantly reduce the facial mechanical pain threshold from the 40^th^ min after injection, and the effect could last to the 220^th^ min (Figure 1C, *n* = 6). Moreover, the expression of CCL2 was increased in the TNC region at the 150^th^ min after TNF-α injection (Figure 1D, ^**^p < 0.01, *n* = 6), but CXCL1 did not (Figure 1D, *n* = 6).

It has been shown that in the condition of TNF-α stimulation or nerve injury, the JNK of astrocytes could be activated to stimulate the secretion of CCL2, which would act on the CCR2 of neurons to elevate neuronal excitability to promote and maintain the neuropathic pain (Lu et al., 2014). We used TNF-α (10 ng/ml) to culture TNC neurons for 1 min and then tested neuronal excitability with whole-cell patch-clamp. The results showed that the TNC neurons cultured with TNF-α produced more action potential spikes significantly than the TNC neurons cultured with a vehicle in the stimulation of double threshold currents (Figures 1E,F, ^**^p < 0.01, *n* = 10). The above data showed that TNF-α might participate in the regulation of TN by promoting the chemokine CCL2 release and elevating the excitability of TNC neurons.

### The Mitogen-Activated Protein Kinases Phosphorylation in the Trigeminal Nucleus Caudalis of Chronic Constriction Injury of Infraorbital Nerve Rats

**FIGURE 2:**
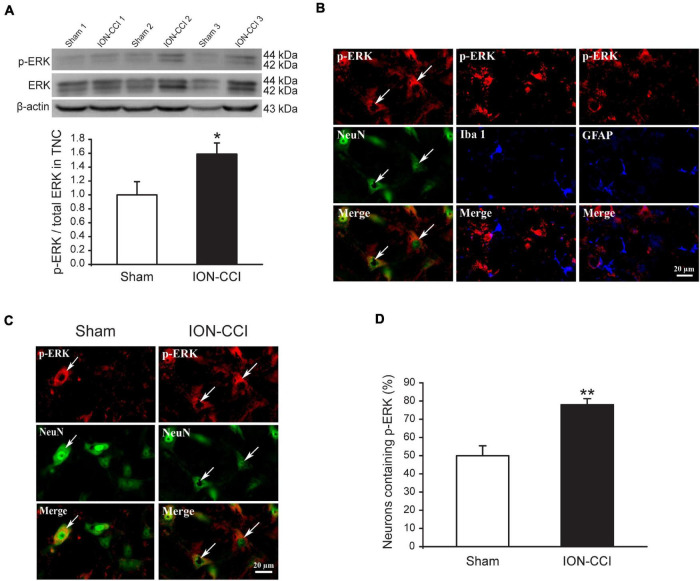
The phosphorylation of ERK is upregulated in the TNC neurons of ION-CCI rats. **(A)** The level of p-ERK in the TNC is increased significantly on the 15th day after ION-CCI operation compared with the sham group,*p < 0.05, *n* = 6. **(B)** The p-ERK is mainly co-localized with NeuN, but not Iba1 or GFAP. **(C,D)** The ratio of TNC neurons of ION-CCI rats that express p-ERK is increased significantly compared with the sham group (77.99 ± 3.34%, *n* = 940 neurons from four rats vs. 49.95 ± 5.43%, *n* = 1,194 neurons from four rats), **p < 0.01. ERK, extracellular signal-regulated kinase; TNC, trigeminal nucleus caudalis; ION-CCI, chronic constriction injury of infraorbital nerve.

**FIGURE 3:**
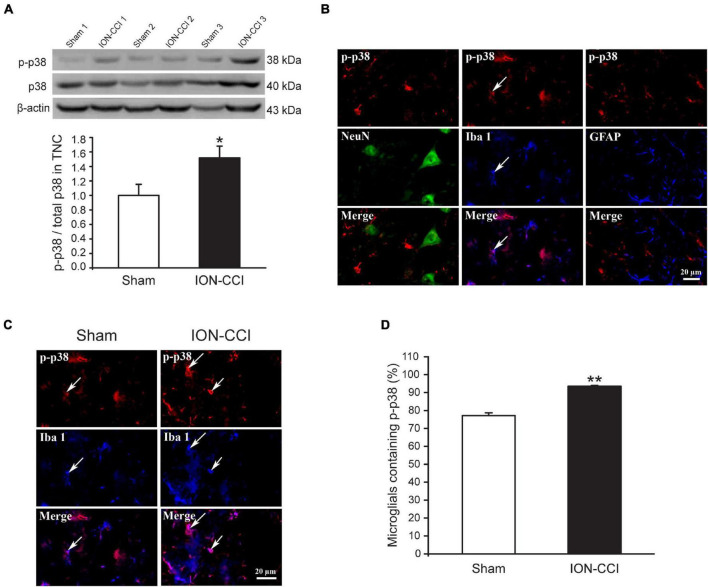
The phosphorylation of p38 is upregulated in the TNC microglia of ION-CCI rats. **(A)** The level of p-p38 in the TNC is increased significantly on the 15th day after the ION-CCI model compared with the sham group, *p < 0.05, *n* = 6. **(B)** The p-p38 is mainly co-localized with Iba1, but not NeuN or GFAP. **(C,D)** The ratio of TNC microglia of ION-CCI rats that express p-p38 is increased significantly compared with the sham group (93.55 ± 0.38%, *n* = 1,424 microglia from 3 rats vs. 77.19 ± 1.54%, *n* = 1,046 microglia from three rats), **p < 0.01. TNC, trigeminal nucleus caudalis; ION-CCI, chronic constriction injury of infraorbital nerve.

**FIGURE 4:**
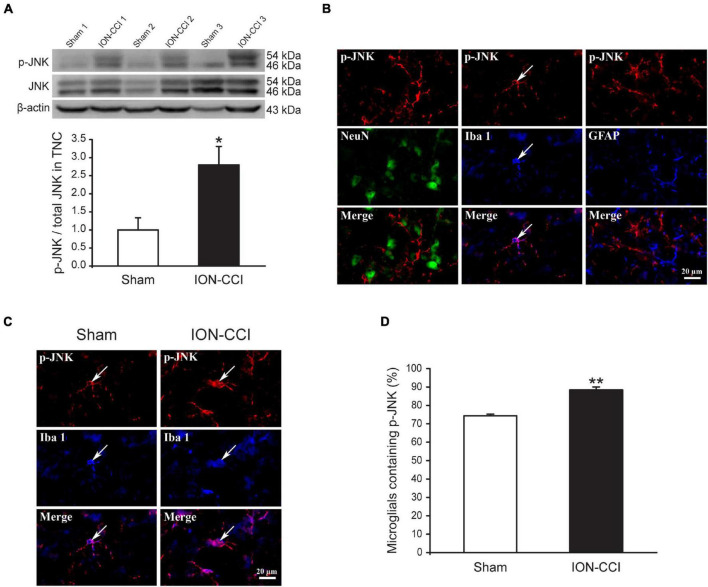
The phosphorylation of JNK is upregulated in the TNC microglia of ION-CCI rats. **(A)** The level of p-JNK in the TNC is increased significantly on the 15th day after the ION-CCI model compared with the sham group, *p < 0.05, *n* = 6. **(B)** The p-JNK is mainly co-localized with Iba1, but not NeuN or GFAP. **(C,D)** The ratio of TNC microglia of ION-CCI rats that express p-JNK is increased significantly compared with the sham group (88.48 ± 1.60%, *n* = 1,337 microglias from three rats vs. 74.35 ± 0.91%, *n* = 1,201 microglia from three rats), **p < 0.01. TNC, trigeminal nucleus caudalis; ION-CCI, chronic constriction injury of infraorbital nerve; jnk, c-Jun N-terminal kinase.

Accumulating evidence has shown that MAPKs (ERK, p38, and JNK) are activated in spinal neurons and glial cells after nerve injury, which contributes to the generation of neuropathic pain (Ji et al., 2002; Jin et al., 2003; Zhuang et al., 2006). In the current study, we examined the phosphorylation level of MAPKs in the TNC of sham and ION-CCI rats by western blotting. On the postoperative day 15, the normalized level of p-ERK in the TNC of ION-CCI rats was increased significantly compared with that of the sham rats (Figure 2A, *p < 0.05, *n* = 6). As shown in Figure 2B, the p-ERK was co-localized with NeuN (marker of neuron) but not with Iba 1 (marker of microglia) or GFAP (marker of astrocyte) in the TNC region. The TNC neurons that expressed p-ERK in the ION-CCI rats were increased significantly compared with those in the sham group (77.99 ± 3.34% vs. 49.95 ± 5.43%) (Figures 2C,D, ^**^p < 0.01). The normalized level of p-p38 and p-JNK was also increased significantly in the TNC of ION-CCI rats (Figures 3A, 4A, *p < 0.05, *n* = 6). Interestingly, the p-p38 and p-JNK were co-localized with Iba1 but not NeuN or GFAP in the TNC region (Figures 3B, 4B). At the same time, the expression of p-p38 in the TNC microglia of ION-CCI rats was also increased significantly compared with the sham group (93.55 ± 0.38% vs. 77.19 ± 1.54%), and so did p-JNK (88.48 ± 1.60% vs. 74.35 ± 0.91%; Figures 3C,D, 4C,D, ^**^p < 0.01).

### The Effects of Mitogen-Activated Protein Kinases Phosphorylation on the Trigeminal Neuralgia

**FIGURE 5:**
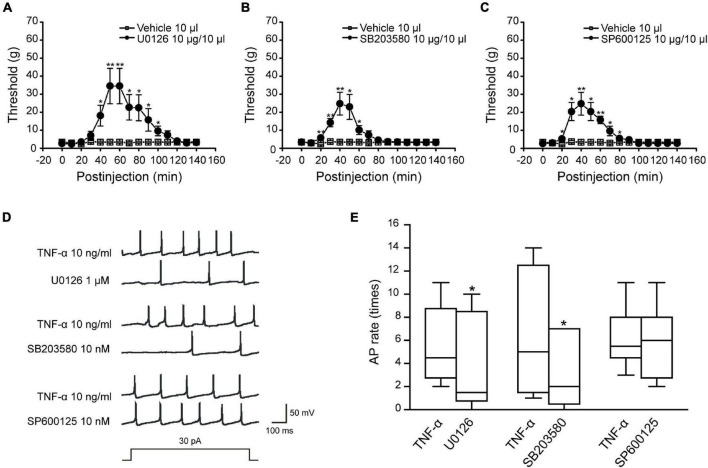
The inhibitors of MAPKs could relieve the facial mechanical pain induced by the ION-CCI model and the activity of MAPKs affects the excitability of TNC neurons induced by TNF-α. **(A–C)** The injection of U0126 (10 μg and 10 μl), SB203580 (10 μg and 10 μl), or SP600125 (10 μg and 10 μl) to the fourth brain ventricle of ION-CCI rats could increase the facial mechanical pain threshold significantly compared with the vehicle (10 μl), *p < 0.05, **p < 0.01, *n* = 6. The increase of facial mechanical pain threshold reaches the maximum at the 50th min after injection of U0126 (34.52 ± 9.82 g), the 40th min after injection of SB203580 (24.74 ± 6.32 g) and SP600125 (24.74 ± 6.32 g). **(D,E)** U0126 (1 μM) and SB203580 (10 nM) pre-incubated for 2 min, respectively, could significantly reduce the frequency of action potential in TNC neurons induced by TNF-α under the double threshold currents stimulation, but SP600125 (10 nM) could not with the same current stimulation and drug-delivery way. *p < 0.05, *n* = 6. TNC, trigeminal nucleus caudalis; ION-CCI, chronic constriction injury of infraorbital nerve; MATKs, mitogen-activated protein kinases.

Previous studies have shown that ION-CCI rats would develop an oral-facial mechanical allodynia after the surgery (Liu et al., 2015). Therefore, we measured the mechanical pain threshold before and after ION-CCI surgery in the same way. The results showed that the oral-facial mechanical pain threshold in the ipsilateral side of ION-CCI rats was decreased significantly compared with that in the sham rats (3.39 ± 0.46 g vs. 58.17 ± 2.83 g; data not shown) on the postoperative day 15. We further explored the role of MAPKs (ERK, p38, and JNK) of the TNC region to the TN using the model rats. The sham and ION-CCI rats were respectively divided into three groups randomly on 15^th^ day, and then got the injection of U0126 (10 μg and 10 μl), SB203580 (10 μg and 10 μl) and SP600125 (10 μg and 10 μl) in the fourth brain ventricle, respectively. The facial mechanical pain threshold was measured before and every 10 min after drug administration. The results showed that all the three inhibitors could significantly increase the facial mechanical pain threshold in the ION-CCI rats, and the maximum effects were around 40–50^th^ min after injection (Figures 5A–C, *p < 0.05, ^**^p < 0.01, *n* = 6). However, all the inhibitors had no effects on the facial mechanical pain threshold in the sham rats (data not shown).

We further researched the effects of MAPKs on the excitability of TNC neurons. The action potential recordings showed that U0126 (1 μM) and SB203580 (10 nM) pre-incubated for 2 min, respectively, could reduce the action potential frequency (Figures 5D,E, ^**^p < 0.01, *n* = 6) but JNK inhibitor SP600125 (10 nM) could not under the double threshold currents stimulation. Combined with the above studies for TNF-α and MAPKs phosphorylation of TNC region in the ION-CCI rats, TNF-α was proposed to increase the phosphorylation of ERK and p38, which might participate in the regulation of neuropathic pain by regulating the excitability of TNC neurons.

### The Expression of BK_*Ca*_ Channels in the Trigeminal Nucleus Caudalis of Chronic Constriction Injury of Infraorbital Nerve Rats

**FIGURE 6:**
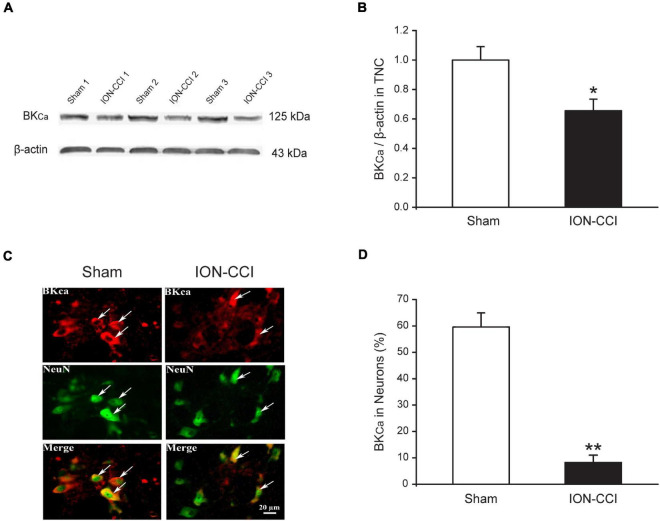
The expression of BK_*Ca*_ channels is reduced in the TNC neurons of ION-CCI rats. **(A,B)** The expression of BK_*Ca*_ channels in TNC of ION-CCI rats is reduced compared with the sham rats, *p < 0.05, *n* = 6. **(C,D)** The BK_*Ca*_ channels are co-localized with the NeuN in the TNC region, and the expression ratio is also reduced significantly in the TNC neurons of ION-CCI rats compared with the sham group (8.25 ± 2.77%, *n* = 1,121 neurons from three rats vs. 59.59 ± 5.34%, *n* = 976 neurons from three rats), **p < 0.01. TNC, trigeminal nucleus caudalis; ION-CCI, chronic constriction injury of infraorbital nerve; BK_*Ca*_, Ca^2+^-activated K^+^ channels.

Our previous study has found that ION-CCI could reduce the expression of BK_*Ca*_ channels in mRNA and protein levels in the ipsilateral trigeminal ganglion (TG). In addition, target injection of NS1619, an opener of BK_*Ca*_ channels, would dose-dependently increase the mechanical pain threshold of ION-CCI rats (Liu et al., 2015). We would like to further investigate whether the expression of BK_*Ca*_ channels in the TNC region was changed in ION-CCI rats by western blotting. The results showed that the expression of BK_*Ca*_ channels in the TNC region from ION-CCI rats was reduced significantly compared with the sham rats (Figures 6A,B, *p < 0.05, *n* = 6). At the same time, we also applied immunofluorescence histochemistry to quantify the BK_*Ca*_ channels, whose expression in the neurons of TNC of ION-CCI rats was decreased tremendously compared with the sham group (8.25 ± 2.77% vs. 59.59 ± 5.34%; Figures 6C,D, ^**^p < 0.01).

### The Effects of Mitogen-Activated Protein Kinases on the Ca^2+^-Activated K^+^ Channels Currents of Trigeminal Nucleus Caudalis Neurons

**FIGURE 7:**
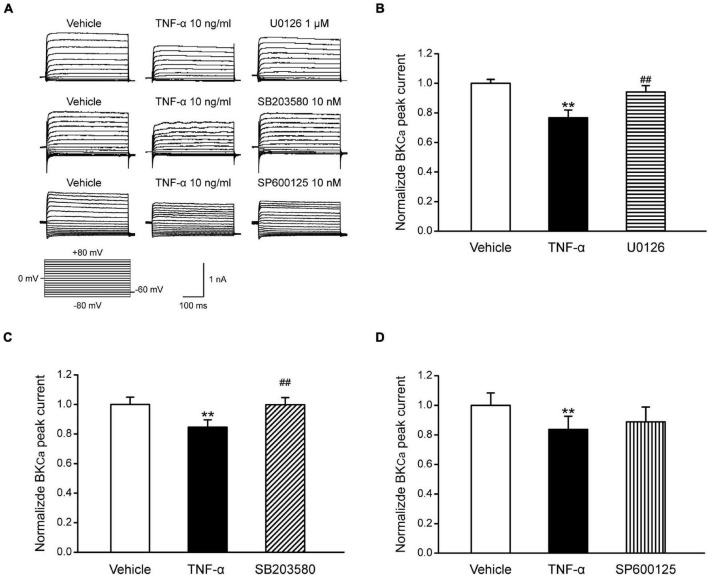
The effects of MAPKs on BK_*Ca*_ currents in TNC neurons of normal rats. **(A)** TNF-α (10 ng/ml) pre-incubated for 1 min, reduces the BK_*Ca*_ peak currents of TNC neurons significantly (**p < 0.01, *n* ≥ 10). U0126 (1 μM) and SB203580 (10 nM) pre-incubated for 2 min, respectively, could largely reverse the effect of TNF-α (^##^*p* < 0.01, n ≥ 10), but SP600125 (10 nM) could not. **(B–D)** The statistical analysis of the data of each group. TNC, trigeminal nucleus caudalis; ION-CCI, chronic constriction injury of infraorbital nerve; BK_*Ca*_, Ca^2+^-activated K^+^ channels.

**FIGURE 8:**
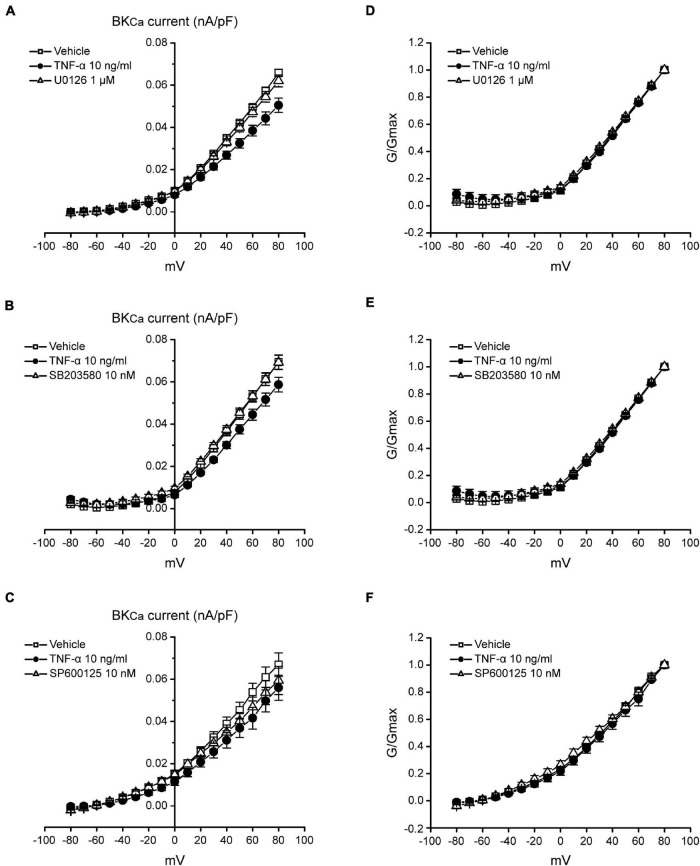
The I-V and G-V curves of BK_*Ca*_ currents in TNC neurons of normal rats. **(A–C)** The current density-voltage curves of BK_*Ca*_ induced by TNF-α could be reversed by U0126 (1 μM) and SB203580 (10 nM), but not SP600125 (10 nM). I–V curve is the currents follows a pre-pulse (0 mV, 100 ms) and a followed 400 ms test pulses from −80 to +80 mV with 10 mV increments. **(D–F)** The G-V curve of BK_*Ca*_ induced by TNF-α is not changed in the presence of U0126, SB203580 or SP600125. G-V curve is the G/Gmax follows a pre-pulse (0 mV and 100 ms) and a followed 400 ms test pulses from −80 to +80 mV with 10 mV increments. TNF-α, tumor necrosis factor alpha; BK_*Ca*_, Ca^2+^-activated K^+^ channels.

Studies have shown that the BK_*Ca*_ channel is an important regulator for neuron excitability (Sah and Faber, 2002; Zhang et al., 2010). NS1619 could reduce the fire of action potentials of dorsal root ganglion (DRG) neurons by activating BK_*Ca*_ channels and further reverse allodynia and hyperalgesia caused by L4–L5 nerve injury (Chen et al., 2009). Our data have illustrated that TNF-α could regulate the excitability of TNC neurons by increasing MAPKs (ERK and p38) phosphorylation and reducing the expression of BK_*Ca*_ channels in the TNC neurons of ION-CCI rats. Therefore, whether BK_*Ca*_ channels are influenced by MAPKs phosphorylation to regulate the excitability of TNC neurons deserves to be studied. In the current study, we found that TNF-α could reduce the BK_*Ca*_ peak currents in the TNC neurons of normal rats significantly (Figures 7A–D), while this effect could be inverted by the ERK inhibitor U0126 (1 μM; Figure 7B, *n* = 11, ^**^p < 0.01) and p38 inhibitor SB203580 (10 nM; Figure 7C, *n* = 10, ^**^p < 0.01), but not JNK inhibitor SP600125 (10 nM; Figure 7D, *n* = 10, *p* > 0.05), which were pre-incubated for 2 min, respectively. It should be pointed that the G-V curves of BK_*Ca*_ induced by TNF-α were not changed in the presence of U0126, SB203580, or SP600125 (Figure 8). The results suggest that the phosphorylation of ERK and p38, which might result in the maintenance of TN, might upregulate the TNC neuron excitability by inhibiting the BK_*Ca*_ currents.

## Discussion

In this study, we showed that: (1) the inflammatory cytokine TNF-α, chemokine CCL2, and CXCL1 are increased significantly in the TNC region of ION-CCI rats. (2) Injecting exogenous TNF-α into the fourth brain ventricle of normal rats could reduce the facial mechanical pain threshold significantly just like the ION-CCI model and promote the expression of CCL2 but not CXCL1 in the TNC region. In addition, TNF-α also increased the fire of action potential of TNC neurons. (3) The phosphorylation of MAPKs (ERK, p38, and JNK) was increased significantly in the TNC region of ION-CCI rats, and target injecting U0126, SB203580, or SP600125 could reverse the facial mechanical allodynia. In addition, the frequency of action potentials of TNC neurons induced by TNF-α could be inhibited by U0126 and SB203580. (4) The expression of BK_*Ca*_ channels was reduced in the TNC region of ION-CCI rats; TNF-α could reduce the BK_*Ca*_ currents in the TNC neurons of normal rats, and the effects could be inhibited by ERK inhibitor U0126 and p38 inhibitor SB203580. These results suggest that the facilitated phosphorylation of ERK and p38 by TNF-α would probably result in the maintenance of TN by inhibiting the expression and function of BK_*Ca*_ channels and enhancing the TNC neuron excitability.

### The Role of TNF-α in the Neuropathic Pain

It has been reported that the proinflammatory cytokines TNF-α, IL-1β, and chemokine CCL2 are upregulated in spinal cord glial cells (Scholz and Woolf, 2007; Ji et al., 2013) and involved in the enhancement of neuropathic pain after the tissue or nerve damage (Gao et al., 2009; Imai et al., 2013). The neuron biopsy with neuropathic pain in some cases of illness also displayed higher expression of TNF-α in Schwann cells (Empl et al., 2001).

In the rat standard model of CCI of the sciatic nerve, TNF-α was detected to be elevated at the injury site (Sommer et al., 1993; Shubayev and Myers, 2000; George et al., 2004), mainly in macrophages (Frisen et al., 1993; Sommer and Schafers, 1998) and Schwann cells (Wagner and Myers, 1996). Incubation of primary culture of astrocytes with TNF-α induced a marked increase in the levels of chemokines, namely, CCL2 and CXCL1, and contributed to central sensitization and chronic pain (Gao et al., 2009). In the present study, TNF-α (20 ng and 10 μl) could significantly decrease the facial mechanical pain threshold, which is consistent with the reports that intra-sciatic injection of TNF-α in rats reproduced pain hypersensitivity (Wagner and Myers, 1996; Sorkin and Doom, 2000). Therefore, our results further confirm that the inflammatory microenvironment and the release of TNF-α are pivotal for the development of neuropathic pain.

In addition, chemokines CCL2 and CXCL1 are increased significantly in the TNC region of ION-CCI rats, implying that they may also be involved in the development and progression of TN in some way, which would still be important research content in the future. Chemokines CCL2 and CXCL1, mainly released by glial cells when nerve injury or neuron inflammation occurs, have the capacity to promote interaction between neurons and glial cells by interacting with their respective receptors in the nervous system, thus contributing to the neuropathic pain process. In the current study, TNF-α injected into the fourth brain ventricle of normal rats could simulate similar facial mechanical pain just like the ION-CCI model rats and increase the expression of CCL2, but the expression of CXCL1 did not change. This may be due to some discrepancy between artificial cerebral ventricle injection of TNF-α and surgical ION-CCI modeling. It has been reported that spinal nerve ligation (SNL) induced persistent neuropathic pain and sustained CXCL1 upregulation in the spinal cord astrocytes. Intrathecal administration of CXCL1 neutralizing antibody transiently reduced SNL-induced pain hypersensitivity (Zhang et al., 2013). These results suggest that the ION-CCI-induced mechanical allodynia in rats probably involves TNF-α and CCL2 pathways in TNC rather than CXCL1. The underlying mechanisms of chemokine CXCL1 in the TNC region from ION-CCI rats require further investigation.

### Mitogen-Activated Protein Kinases Phosphorylation Participates in the Regulation of Neuropathic Pain

The MAPK family mainly contains ERK, p38, and JNK, representing three different signal transduction pathways. Increasing evidence shows that MAPKs in spinal glial cells and neurons play important roles in the induction and maintenance of chronic pain and neuropathic pain (Ji et al., 2002; Jin et al., 2003; Ji and Strichartz, 2004; Zhuang et al., 2006). After nerves injury, the activation of p-ERK in spinal microglia corresponds to the early phase and then gradually transitions to astrocytes in the late phase and participates in the regulation of neuropathic pain (Zhuang et al., 2005; Wang et al., 2012). When inflammation occurs, the ERK activation of spinal neurons would be increased, contributing to the pathogenesis of inflammatory pain (Cao et al., 2014). The phosphorylation of p38 increases in spinal cord microglia after nerve injury (Tsuda et al., 2004; Kobayashi et al., 2008), spinal cord injury (Hains and Waxman, 2006), acute inflammation induced by formalin (Svensson et al., 2003), surgery-evoked postoperative pain (Wen et al., 2009; Peters and Eisenach, 2010), and chronic opioid exposure (Cui et al., 2006), serving as a key signaling molecule in microglia by integrating various inputs to microglia (Ji and Strichartz, 2004). Intrathecal inhibition of p38 has been shown to attenuate neuropathic pain in different animal models (Schafers et al., 2003; Sweitzer et al., 2004). Some studies have reported that JNK is activated in DRG neurons and spinal astrocytes after nerve injury, whereas transient activation of JNK in DRG neurons is involved in the induction of neuropathic pain, and persistent activation of JNK in spinal astrocytes appears critical for the maintenance of neuropathic pain. After TNF-α stimulation and nerve injury, JNK would be activated in spinal cord astrocytes, which is critical for the development and maintenance of neuropathic pain (Zhuang et al., 2006; Gao et al., 2009). Therefore, inhibiting the phosphorylation of ERK, p38, and JNK can be effective to alleviate inflammatory and neuropathic pain (Manassero et al., 2012; Lu et al., 2013).

Our previous study has shown that MAPKs (ERK, p38, and JNK) in the TG neurons of rats participated in the regulation of trigeminal neuropathic pain (Liu et al., 2015). Therefore, this study is further to research the MAPKs regulation mechanism in the TNC of the ION-CCI rats. Our results demonstrate that ERK1/2, p38, and JNK inhibitors all significantly reversed the facial mechanical allodynia induced by ION-CCI. The level of p-ERK was increased significantly in TNC neuron, and so were p-p38 and p-JNK in TNC microglia after ION-CCI operation. The inhibitor of ERK or p-38 could reduce the firing frequency of action potentials induced by TNF-α, suggesting the involvement of the activation of p38 and JNK in TNC microglia in ION-CCI rats. They may contribute to the increase of the excitability of TNC neurons by phosphorylating MAPK kinases directly or indirectly, further inducing neuropathic pain.

### Ca^2+^-Activated K^+^ Channels Are Affected by Mitogen-Activated Protein Kinases Phosphorylation in the Neuropathic Pain

Large conductance Ca^2+^-activated K channels (BK_*Ca*_ channels) are characterized by a large single-channel conductance (> 100 pS) and expressed in sensory neurons, which are activated by the synergistic interaction of elevated intracellular Ca^2+^ and electrical depolarization of the membrane to cause rapid efflux of K^+^ (Rothberg, 2012). BK_*Ca*_ channel belongs to the key regulator of neuronal excitability by exerting inhibitory control on sensory input in inflammatory pain states (Sah and Faber, 2002; Zhang et al., 2010). Some studies have shown that NS1619 could reduce the depolarization-evoked action potential firing of DRG neurons (Zhang et al., 2003) and the allodynia and hyperalgesia caused by L4-L5 nerve injury by opening BK_*Ca*_ channels (Chen et al., 2009). IbTX, an inhibitor of the BK_*Ca*_ channel, is able to increase the sEPSCs of the superficial dorsal horn when administered presynaptically and modulate the nociceptive transmission (Furukawa et al., 2008). Our previous study has shown that both of the expression and function of BK_*Ca*_ channels would be downregulated in the ipsilateral TG of the ION-CCI model rats. NS1619 could increase the mean threshold intensities of action potentials and the mechanical pain threshold in ION-CCI rats (Liu et al., 2015). The TG and the TNC are important structures in migraine pathophysiology given that all painful trigeminal sensations are conveyed by the TG to the TNC, which further transmit nociceptive signals to the sensory cortex (DeLeo et al., 2004). However, whether BK_*Ca*_ channels have a similar effect in the TNC region as in the TG neurons is still unknown. In this study, we showed that the expression of BK_*Ca*_ channels was decreased significantly in the TNC region of ION-CCI model rats. Meanwhile, U0126 and SB203580, the inhibitor of ERK and p-38 respectively, could reverse the decrease of BK_*Ca*_ currents induced by TNF-α in the TNC neurons of normal rats. These results demonstrate that the phosphorylation of ERK and p38 is involved in the regulation of BK_*Ca*_ channels by TNF-α and further affects the regulation of trigeminal neuropathic pain.

Taken together, these results demonstrate that TNF-α could increase the phosphorylation of ERK and p38, which would result in the maintenance of TN by inhibiting the BK_*Ca*_ currents and upregulating the TNC neuron excitability. TNF-α has been reported to increase the frequency and amplitude of Ca^2+^ spark-induced large conductance Ca^2+^-activated potassium (BK_*Ca*_) channel transients to 1.7- and 1.4-fold in smooth muscle cells, respectively. Furthermore, data indicate that TNF-α activates NAD(P)H oxidase, resulting in an increase in intracellular H_2_O_2_ that stimulates Ca^2+^ sparks and transient BK_*Ca*_ currents, leading to a reduction in global [Ca^2+^]_*i*_ and vasodilation (Cheranov and Jaggar, 2006). In the present study, the very quick effects (in 1 min) of TNF-α suggest the direct effect of on channel protein or changes in local Ca concentration in the vicinity of the channel due to reactive oxygen species (ROS) production, which needs further investigation.

## Data Availability Statement

The original contributions presented in the study are included in the article/Supplementary Material, further inquiries can be directed to the corresponding author/s.

## Ethics Statement

The animal study was reviewed and approved by Naval Medical University Institutional Animal Care and Use Committee.

## Author Contributions

ZL: ION-CCI and sham operation, behavior testing, western blotting analysis, immunofluorescence histochemistry, whole-cell patch-clamp recording, and wrote the manuscript. JF: behavior testing, ELISA, and data collection and analysis. LY: data collection. BM: the study and designed specific tests. LC: specific tests. All authors contributed to the article and approved the submitted version.

## Conflict of Interest

The authors declare that the research was conducted in the absence of any commercial or financial relationships that could be construed as a potential conflict of interest.

## Publisher’s Note

All claims expressed in this article are solely those of the authors and do not necessarily represent those of their affiliated organizations, or those of the publisher, the editors and the reviewers. Any product that may be evaluated in this article, or claim that may be made by its manufacturer, is not guaranteed or endorsed by the publisher.
