# Supplementary material for: The Up-regulation of TNF-α Maintains Trigeminal Neuralgia by Modulating MAPKs Phosphorylation and BKCa Channels in Trigeminal Nucleus Caudalis
Source: Front Cell Neurosci. 2021 Nov 25;15:764141. doi: 10.3389/fncel.2021.764141 (PMC8657151; doi:10.3389/fncel.2021.764141)
Supplement: Supplementary file 1 [file Data_Sheet_1.PDF]

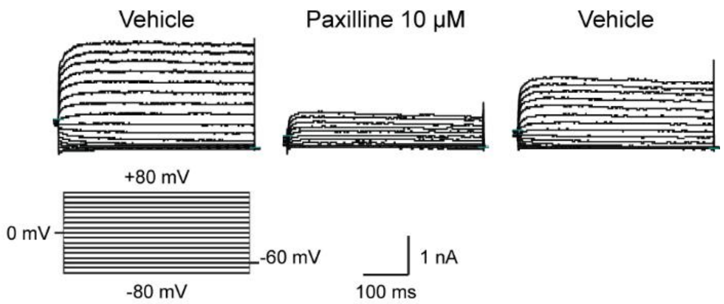

S-Figure 1 The effects of paxilline on BK_{Ca} currents in TNC neurons of normal rats. (A) Paxilline, a specific antagonist of BK_{Ca} channel, reduces the BK_{Ca} peak currents of TNC neurons significantly when pre-incubated for 2 minutes with the concentration of 10 μM (n=14, in 9 normal rats).

S-Table 1. Properties of TNC neurons.

	RMP (mV)	Capacitance (pF)	Threshold current (pA)	Duration (ms)	AP amplitude (mV)	fAHP (mV)
Vehicle	-53.25±1.72	34.4±1.57	15.83±1.58	5.68±0.34	49.51±1.42	17.62±0.94

TNC: trigeminal nucleus caudalis; RMP: rest membrane potential; fAHP: fast after-hyperpolarization (n=24, in 11normal rats).
